# Reductive C(sp^3^)–C(sp^3^) homo-coupling of benzyl or allyl halides with H_2_ using a water-soluble electron storage catalyst†

**DOI:** 10.1039/d1ra08596d

**Published:** 2021-12-09

**Authors:** Takeshi Yatabe, Sayaka Futakuchi, Keishi Miyazawa, Daiki Shimauchi, Yukina Takahashi, Ki-Seok Yoon, Hidetaka Nakai, Seiji Ogo

**Affiliations:** Department of Chemistry and Biochemistry, Graduate School of Engineering, Kyushu University 744 Moto-oka, Nishi-ku Fukuoka 819-0395 Japan ogo.seiji.872@m.kyushu-u.ac.jp; International Institute for Carbon-Neutral Energy Research (WPI-I2CNER), Kyushu University 744 Moto-oka, Nishi-ku Fukuoka 819-0395 Japan; Center for Small Molecule Energy, Kyushu University 744 Moto-oka, Nishi-ku Fukuoka 819-0395 Japan; Department of Applied Chemistry, Faculty of Science and Engineering, Kindai University 3-4-1 Kowakae, Higashi-Osaka Osaka 577-8502 Japan

## Abstract

This paper reports the first example of a reductive C(sp^3^)–C(sp^3^) homo-coupling of benzyl/allyl halides in aqueous solution by using H_2_ as an electron source {turnover numbers (TONs) = 0.5–2.3 for 12 h}. This homo-coupling reaction, promoted by visible light, is catalysed by a water-soluble electron storage catalyst (ESC). The reaction mechanism, and four requirements to make it possible, are also described.

## Abstract 2

Four requirements for the reductive C(sp^3^)–C(sp^3^) homo-coupling of benzyl/allyl halides in aqueous solution by using H_2_ as an electron source are described.

## Graphical abstract

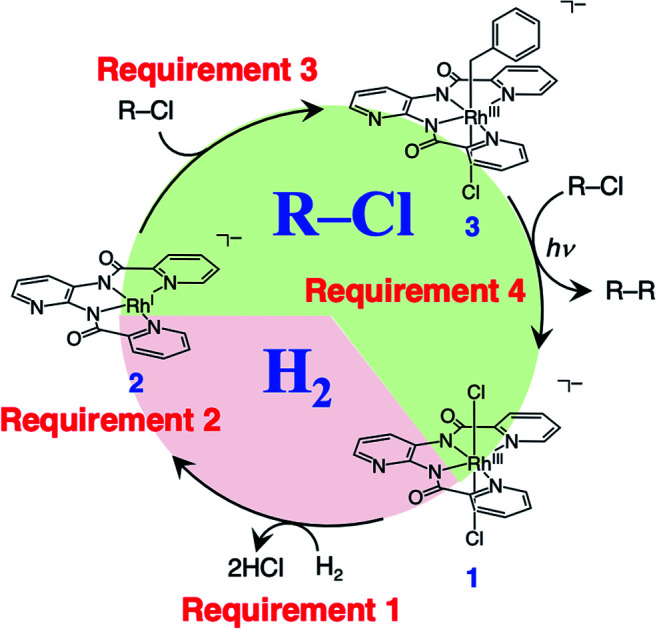

C–C bond formation reactions, such as reductive coupling, oxidative coupling, C–H arylation and cross coupling, are very important tools in organic synthesis. Various types of reactions and combinations of electrophiles, nucleophiles, reducing agents, oxidants, *etc.* have been investigated in search of selective, efficient, mild and environmentally friendly C–C bond formation reactions.^1^

Metals such as Na, Mn, Cu and Zn have been widely used as reductants in such C–C bond formation reactions (Table S1†).^1*b*,2^ However, these materials often cause unwanted side-reactions and their disposal is costly, environmentally destructive or both.

The use of H_2_ as a reducing agent, therefore, has many potential advantages over these metals.^3^ One particular advantage is that it is a relatively unreactive gas, meaning it is unlikely to participate in unwanted side-reactions, but this lack of reactivity cuts both ways. Without a catalyst to assist the reaction, H_2_ is very slow to give up its electrons for reduction. To this end, we have been developing a range of catalysts that bind to H_2_ and store its electrons for employment in reduction. Here, we report the use of such a catalyst in the reductive C(sp^3^)–C(sp^3^) homo-coupling of benzyl or allyl halides, promoted by visible light.

An example of reductive C(sp^3^)–C(sp^3^) homo-coupling of allylamines or allylic alcohols using H_2_ has recently been reported by Huang and coworkers.^3*b*^ However, although it is an important development in the field, its employment is somewhat limited and no reaction mechanism has been identified. We have chosen benzyl or allyl halides and have conducted extensive investigations to elucidate not only the mechanism of our reaction but four necessary requirements for proper reactivity. The reactions are centred on derivatives of our successful [NiFe]hydrogenase-mimic catalysts, which have previously been employed as electron storage catalysts (ESCs) in hydrogen fuel cells and direct synthesis of H_2_O_2_.^4^

Combining our previous studies with those of this research, we determined that the following four requirements would be required to perform reductive homo-coupling or C–H arylation with an ESC. Requirement 1: if we want to use electrons from H_2_, we should perform the reaction in water. Primarily, this motivation arises from the considerable environmental benefits of omitting organic solvents, but the heterolytic cleavage of H_2_ is also favourable in water. Requirement 2: an electron-withdrawing effect from the ligand helps to store the electrons from H_2_ on the metal centre. Requirement 3: the catalyst requires vacant coordination sites where R and X are oxidatively added to the metal centre after the R–X bond is cleaved. Requirement 4: (1) in the case of reductive homo-coupling, an electron-donating effect from the ligand is required for benzyl/allyl radical transfer *via* M–C bond cleavage. (2) In the case of C–H arylation, aryl radical transfer *via* M–C bond cleavage requires an electron-withdrawing effect from the ligand.

These strategies allowed us to previously develop an ESC, [Rh^III^(L)(Cl)_3_(DMF)] (L = 2,9-dibutyl-1,10-phenanthroline, DMF = *N*,*N*-dimethylformamide) that enables C–H arylation using H_2_ as an electron source under mild conditions.^3*a*^ However, since the necessary requirements for C–H arylation are opposite to those for reductive homo-coupling, this ESC was unable to perform reductive C(sp^3^)–C(sp^3^) homo-coupling of benzyl or allyl halides using H_2_ as an electron source.

In this paper, to solve this problem, we have designed a new ESC, [H^+^][Rh^III^(X)(Cl)_2_] {[H^+^][1], X = *N*,*N*′-bis(2-pyridinecarboxamidato)-2,3-pyridine} with an electron-donating amide group. As a result, the expected benzyl or allyl radical transfer was promoted by this ESC in the presence of photo-irradiation, and we can report the first example of a photoinduced reductive C(sp^3^)–C(sp^3^) homo-coupling reaction of benzyl or allyl halides using H_2_ as an electron source. Our report begins with the synthesis and structural analysis of the ESC. Finally, we report on stoichiometric and catalytic reactions using various benzyl/allyl halides substrates.

The ESC, [H^+^][1], was synthesised by the reaction of Rh^III^Cl_3_ with *N*,*N*′-bis(2-pyridinecarboxamide)-2,3-pyridine in DMF at 100 °C for 1 h and was characterised by X-ray analysis (Fig. 1), ^1^H NMR spectroscopy (Fig. S1†), electrospray ionisation-mass spectrometry (ESI-MS, Fig. S2†), X-ray powder diffraction analysis (Fig. S3†) and elemental analysis. A single crystal of [Ph_4_P^+^][1] suitable for X-ray analysis was obtained by replacing the H^+^ with Ph_4_P^+^ ion. An ORTEP drawing of 1 shows that the Rh^III^ metal centre adopts distorted octahedral geometry with one ligand X and two Cl^−^ ions (Fig. 1). The distances of the Rh–N bonds {1.962(3) and 2.074(3) Å} were comparable to those of previously reported Rh^III^ amide complexes.^5^ The ^1^H NMR spectrum of 1 shows the signals at 7.4–9.5 ppm and at 14.6 ppm derived from ligand X and the proton, respectively (Fig. S1†). The negative-ion ESI mass spectrum of 1 exhibits a prominent signal at *m*/*z* = 490.0 (relative intensity = 100% in the range of *m*/*z* = 200 to 2000). The signal has a characteristic isotopic distribution that matches well with the calculated isotopic distribution for [1]^−^ (Fig. S2†).

**Fig. 1:**
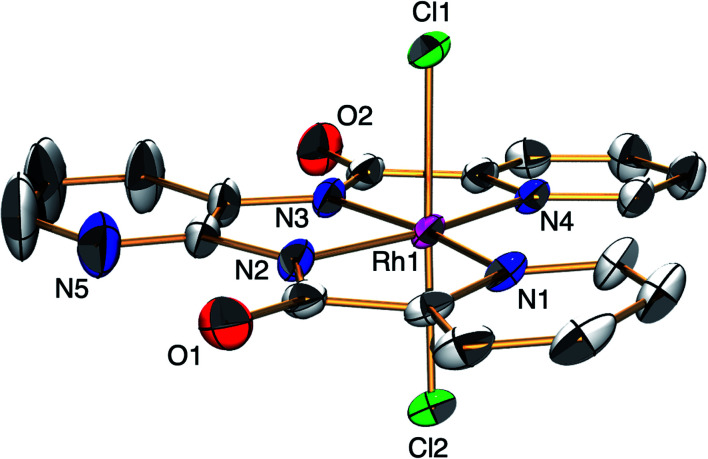
ORTEP drawing of [PPh_4_^+^][1] with the ellipsoids at 50% probability. Countercation and hydrogen atoms are omitted for clarity. Selected interatomic distances (*l*/Å): Rh1–Cl1 = 2.3306(8), Rh1–Cl2 = 2.3394(9), Rh1–N1 = 2.074(3), Rh1–N2 = 1.962(3), Rh1–N3 = 1.962(3), Rh1–N4 = 2.074(3).

[H^+^][1] reacts with H_2_ (0.1–0.9 MPa) to form low-valent Rh^I^ complex [H^+^][Rh^I^(X)] {[H^+^][2]} at 60 °C in H_2_O after 8 h (eqn (1)). Using water as part of the solvent means it can act as a Lewis base to abstract two electrons from H_2_, whereupon they are stored on the Rh centre. This behaviour accounts for Requirement 1 and the electron-withdrawing effect of the ligand X to stabilise the low-valent Rh^I^ centre accounts for Requirement 2. Characterisation of 2 was conducted with ESI-MS (Fig. 2), ultraviolet visible near-infrared (UV-vis-NIR) absorption spectroscopy (Fig. S4†), X-ray photoelectron spectroscopy (XPS, Fig. S5†) and elemental analysis. The positive-ion ESI mass spectrum of 2 indicates a prominent signal at *m*/*z* = 421.9 (relative intensity = 100% in the range of *m*/*z* = 200 to 2000) that has a characteristic isotopic distribution that matches well with the calculated isotopic distribution for [2 + 2H]^+^ (Fig. 2). The UV-vis-NIR absorption spectrum of 2 shows absorption bands at 500–1200 nm, which are assigned to metal-to-ligand charge transfer and metal–metal-to-ligand charge transfer bands and is similar to other Rh^I^ complexes with the polypyridyl ligand (Fig. S4†).^6^ The XPS spectrum of 2 exhibits Rh 3d_3/2_ and Rh 3d_5/2_ peaks at 311.9 and 307.2 eV, which are lower than those of Rh^III^ complex 1 (313.9 and 309.3 eV) and are similar to the other Rh^I^ complexes (Fig. S5†).^3*a*,7^ These results indicate that the oxidation state of Rh in 2 is univalent.1
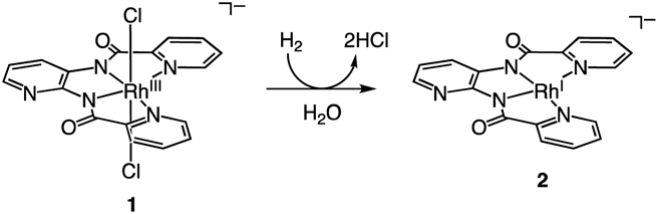


**Fig. 2:**
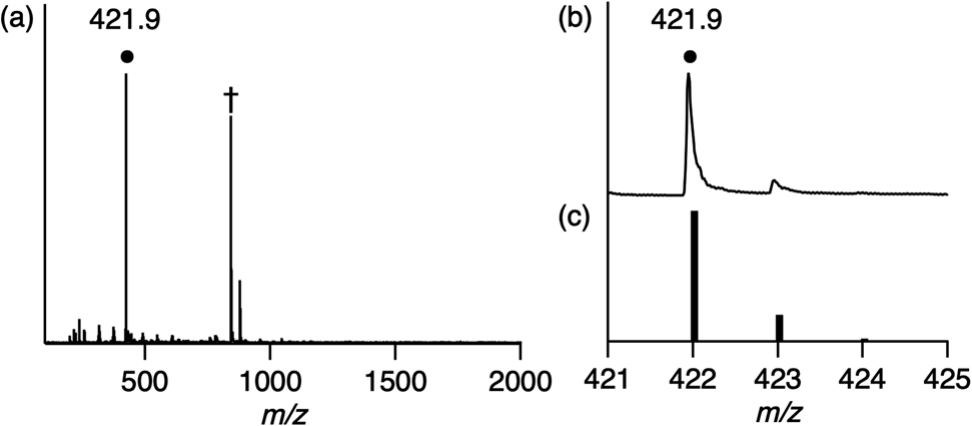
(a) Positive-ion ESI mass spectrum of 2 in methanol. The signal at *m*/*z* = 421.9 corresponds to [2 + 2H]^+^. ^†^The signal at *m*/*z* = 842.9 corresponds to [22 + 3H]^+^. (b) The signal at *m*/*z* = 421.9 for [2 + 2H]^+^. (c) The calculated isotopic distribution for [2 + 2H]^+^.

[H^+^][2] cleaves the C–Cl bond of benzyl chloride to form Rh^III^ complex [Na^+^][Rh^III^(X)(CH_2_C_6_H_5_)(Cl)] {[Na^+^][3]} (eqn (2)). This oxidative addition of benzyl chloride to Rh^I^ indicates that the Rh catalyst transfers the two electrons originally acquired from H_2_ to benzyl chloride by making use of vacant sites on the Rh centre, following Requirement 3. The structure of 3 was elucidated by X-ray analysis (Fig. 3), ESI-MS (Fig. S6†), ^1^H NMR spectroscopy (Fig. S7†), UV-vis-NIR absorption spectroscopy (Fig. S8†) and elemental analysis. A single crystal of 3 suitable for X-ray analysis was obtained by the slow vapor diffusion of diethyl ether into DMF/acetonitrile solution. An ORTEP drawing of 3 shows that the Rh^III^ metal centre adopts distorted octahedral geometry with one ligand X, one benzyl group and one Cl^−^ ion (Fig. 3). The distance of Rh–C(benzyl) (2.094(3) Å) is similar to the Rh–C bonds in other Rh benzyl or allyl complexes (2.078(2)–2.120(4) Å).^8^ The positive-ion ESI mass spectrum of 3 indicates a prominent signal at *m*/*z* = 512.0 (relative intensity = 100% in the range of *m*/*z* = 200 to 2000) (Fig. S6†). This signal has a characteristic isotopic distribution that matches well with the calculated isotopic distribution for [3 − Cl + H]^+^. The ^1^H NMR spectrum of 3 shows the signals at 3.21, 3.25 and 6.3–9.0 ppm, derived from the benzyl group and ligand X (Fig. S7†). The signals at 3.21 and 3.25 are double doublet peaks with coupling constants of 3.6 and 8.4 Hz. This is the expected pattern arising from the geminal coupling and the spin–spin interaction of the methylene protons with the Rh^III^ centre that also possesses a nuclear spin of 1/2. The UV-vis-NIR spectra showed that the absorption bands of 2 at 500–1200 nm disappeared and the characteristic bands of 3 appeared by the reaction of 2 with benzyl chloride (Fig. S8†).2
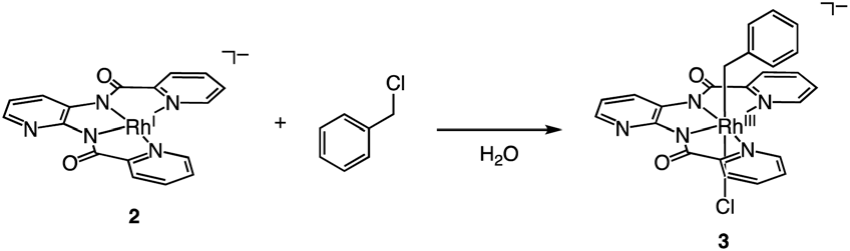


**Fig. 3:**
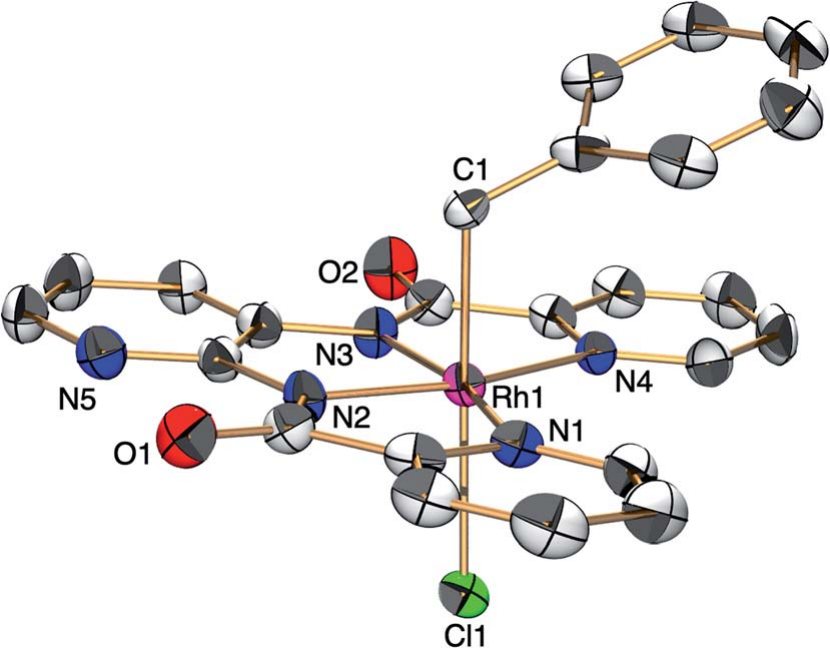
ORTEP drawing of [H^+^][3] with the ellipsoids at 50% probability. Hydrogen atoms are omitted for clarity. Selected interatomic distances (*l*/Å): Rh1–C1 = 2.094(3), Rh1–Cl1 = 2.5480(9), Rh1–N1 = 2.092(3), Rh1–N2 = 1.966(3), Rh1–N3 = 1.973(3), Rh1–N4 = 2.084(3).

Photo-irradiation of the methanol solution of 3 in the presence of benzyl chloride led to the formation of complex 1 and bibenzyl (Fig. S9†). This reaction was monitored by UV-vis-NIR absorption spectroscopy (Fig. S10†), and the detection of 1 was conducted by ESI-MS (Fig. S11†). The absorption spectrum of 3 loses the absorption band at 375 nm, instead exhibiting an absorption band at 400 nm (Fig. S10†). The negative-ion ESI mass spectrum of the reaction solution shows a prominent peak at *m*/*z* = 489.9 (relative intensity = 100% in the range of *m*/*z* = 200 to 2000) and a characteristic distribution that matches well with the calculated distribution of [1]^−^ (Fig. S11†). The yield of bibenzyl was quantified by gas chromatography-mass spectrometry (GC-MS) and determined as 34% based on 3. No bibenzyl was formed from the reaction performed in the dark. Addition of the radical trapping reagent (*N-tert*-butyl-α-phenylnitrone) into the reaction solution decreased the yield of bibenzyl to 5%. These results suggest that the photo-irradiation of 3 led to cleavage of the Rh–C bond, producing the benzyl radical, followed by the radical coupling of the benzyl radical with benzyl chloride to form bibenzyl (Fig. S12†). The remaining chloride radical then bound to the Rh^II^ metal centre to yield complex 1 (Fig. S12†). Similar photoinduced Rh–C bond cleavage has been reported using Rh porphyrin complexes.^9^ Homolytic cleavage of the Rh–R bond led to the generation of a benzyl or allyl radical (R˙) that formed the C(sp^3^)–C(sp^3^) bond with another molecule of benzyl or allyl chloride. Altogether, this process means that the employment of electrons from H_2_ can activate benzyl or allyl chlorides and form benzyl or allyl radicals (R˙) with photo-irradiation.

Having established the stoichiometric reactions, we examined the catalytic homo-coupling reaction of benzyl chloride derivatives or allyl chloride derivatives by 1 under a H_2_ atmosphere and photo-irradiation (Fig. S13† and Table 1). The products were identified by both ^1^H NMR and GC-MS. The isolated yields of the coupling products were measured by a balance. Reductive homo-coupling reactions of benzyl chloride derivatives or allyl chloride derivatives were performed using 1 at 80 °C under a H_2_ atmosphere (0.9 MPa) for 12 h in ethanol/H_2_O (eqn (3) and Table 1, entries 1–6). After the catalytic reaction, 2 was formed because the benzyl chloride derivatives or allyl chloride derivatives were consumed. Although the turnover numbers (TONs) were low (TONs = 0.5–2.3) (entries 1–6), the catalytic reaction must have proceeded by means of 1, H_2_ and photo-irradiation because no homo-coupling products were formed without 1, H_2_ or photo-irradiation (entries 7–9).3
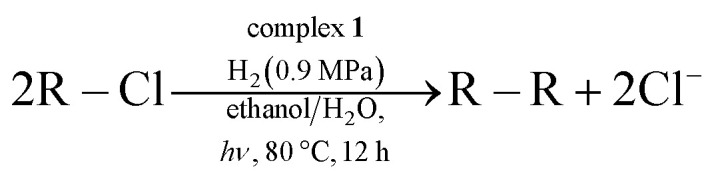


**Table 1:** Photoinduced reductive C(sp^3^)–C(sp^3^) homo-coupling of benzyl chloride derivatives or allyl chloride derivatives (R–Cl) with H_2_ using 1 as the ESC^a^

Entry	R–Cl	Product	TON^b^	Yield^c^
1	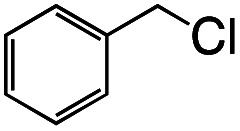	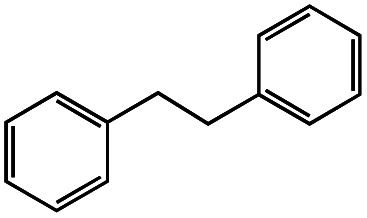	1.7	33
2	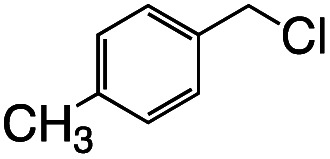	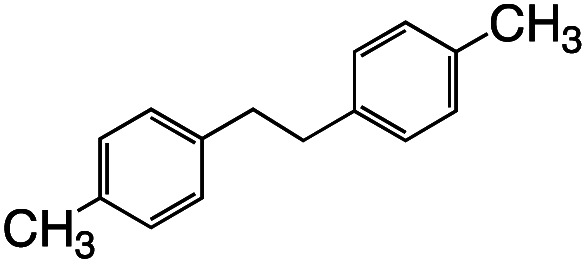	1.5	30
3	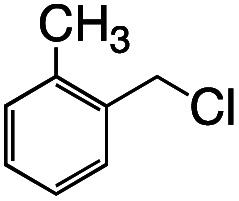	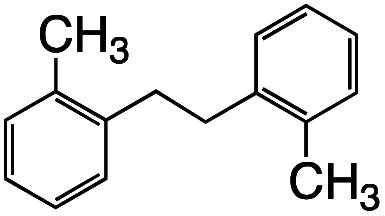	1.4	28
4	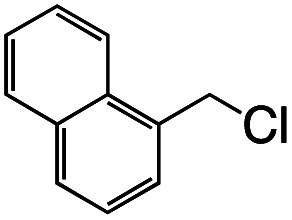	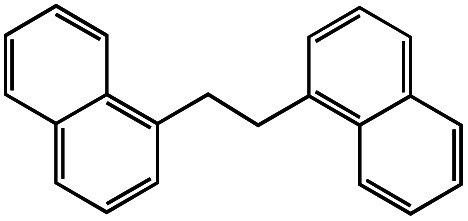	1.7	34
5^d^	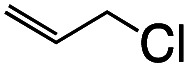	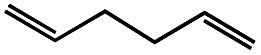	2.3	45
6	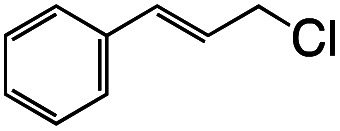	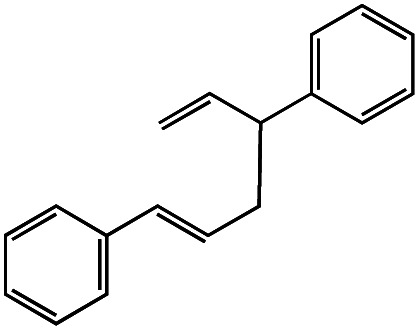	0.3	5.6
		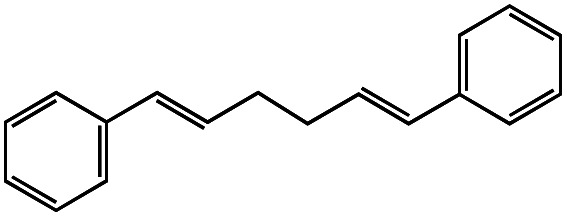	0.2	3.9
7^e^	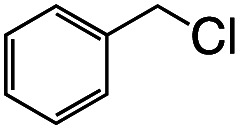	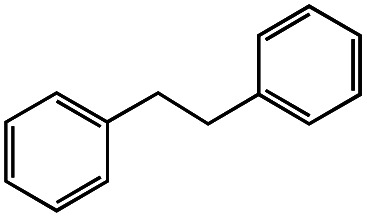	0	0
8^f^	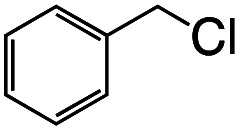	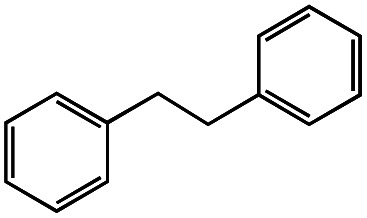	0	0
9^g^	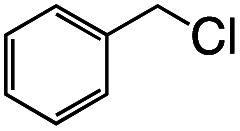	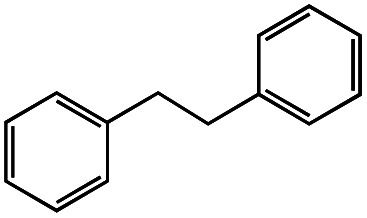	0	0

a Reaction conditions: complex 1 (51 μmol), benzyl chloride derivatives or allyl chloride (0.51 mmol), sodium acetate (417 mg, 5.1 mmol), ethanol (50 mL), H_2_O (51 μmol), 80 °C, 12 h under a H_2_ atmosphere (0.9 MPa) with photo-irradiation (400–800 nm).

b The turnover numbers (TONs, [(mol of coupling product)/(mol of catalyst)]) were determined based on 1.

c The coupling products were identified by both ^1^H NMR and GC-MS. The isolated yields of the products were measured by a balance.

d The yield of 1,5-hexadiene was determined by GC-MS due to the low boiling point of 1,5-hexadiene.

e Reaction was performed without 1.

f Reaction was performed without H_2_.

g Reaction was performed without photo-irradiation.

Based on the above results, we propose the reaction mechanism as shown in Fig. 4. The Rh^III^ complex 1 reacts with H_2_ to form the Rh^I^ complex 2. Oxidative addition of benzyl or allyl chloride to 2 yields the Rh^III^ complex 3. Photo-irradiation for 3 forms the benzyl or allyl radical, which reacts with another benzyl or allyl chloride to afford homo-coupling products. The reaction of Rh^II^ species and chloride radical recovers Rh^III^ complex 1.

**Fig. 4:**
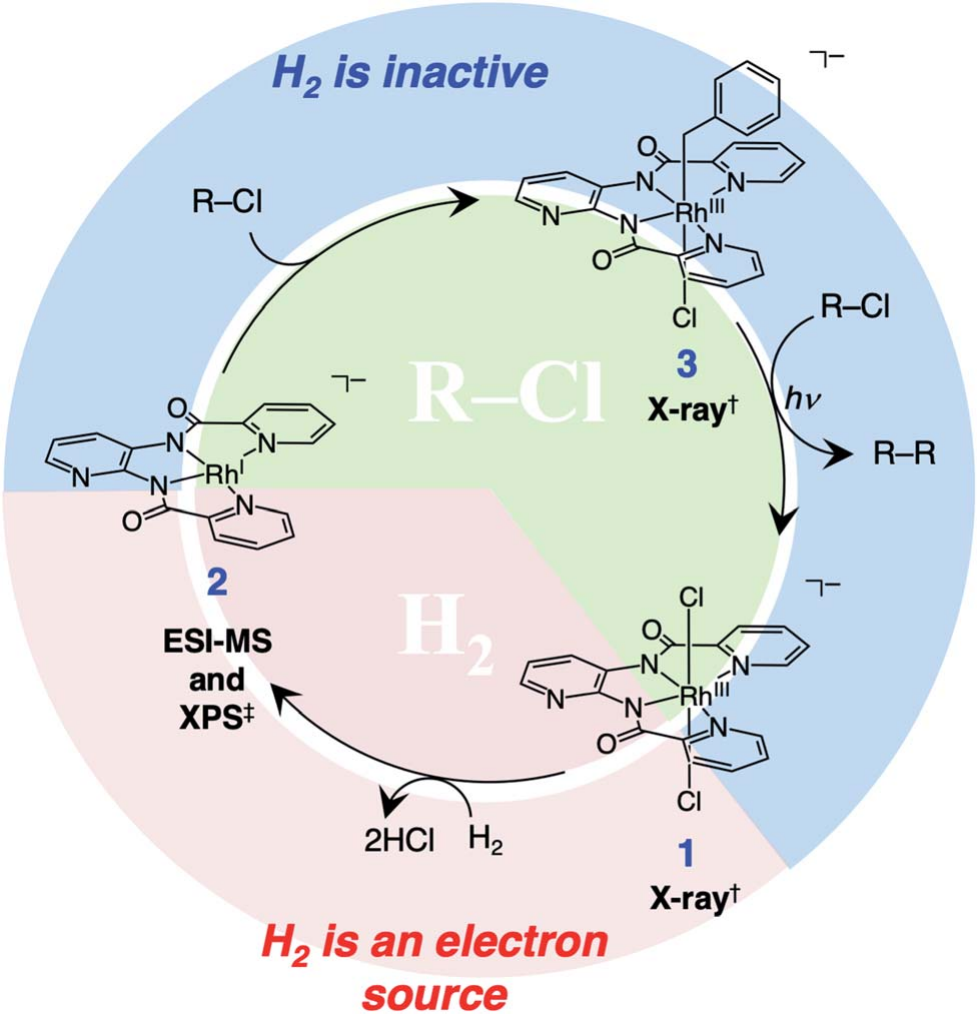
A proposed mechanism of the photoinduced reductive homo-coupling of benzyl or allyl halides with H_2_ catalysed by the ESC. ^†^The structures of 1 and 3 were determined by X-ray analysis. ^‡^2 was characterised by ESI-MS and XPS.

In conclusion, we have reported the photoinduced reductive C(sp^3^)–C(sp^3^) homo-coupling reaction of benzyl or allyl halides in aqueous solution by using the ESC and H_2_. Although the TON of this catalytic reaction is not high, the catalytic mechanism discussed here should provide valuable insights into the development of new ESCs to facilitate the C–C bond formation reaction using H_2_ as an electron source.

## Conflicts of interest

There are no conflicts to declare.

## Supplementary Material

RA-011-D1RA08596D-s001

RA-011-D1RA08596D-s002
