# Supplementary material for: Reductive C(sp3)–C(sp3) homo-coupling of benzyl or allyl halides with H2 using a water-soluble electron storage catalyst
Source: RSC Adv. 2021 Dec 9;11(62):39450–4. doi: 10.1039/d1ra08596d (PMC9044531; doi:10.1039/d1ra08596d)
Supplement: RA-011-D1RA08596D-s001 [file RA-011-D1RA08596D-s001.pdf]

Electronic Supplementary Material (ESI) for RSC Advances.
This journal is © The Royal Society of Chemistry 2021

Electronic Supplementary Information for

Reductive C(sp^3)–C(sp^3) Homo-Coupling of Benzyl or Allyl Halides with H_2 Using a Water-Soluble Electron Storage Catalyst

Takeshi Yatabe, Sayaka Futakuchi, Keishi Miyazawa, Daiki Shimauchi, Yukina Takahashi, Ki-Seok Yoon, Hidetaka Nakai and Seiji Ogo*

*To whom correspondence should be addressed.
E-mail: ogo.seiji.872@m.kyushu-u.ac.jp

This PDF file includes:

Materials and Methods
Figs. S1 to S13
Tables S1 and S2
References

A Table of Contents

Materials and Methods	page 1
Table S1	page 7
Table S2	page 8
Fig. S1	page 9
Fig. S2	page 10
Fig. S3	page 11
Fig. S4	page 12
Fig. S5	page 13
Fig. S6	page 14
Fig. S7	page 15
Fig. S8	page 16
Fig. S9	page 17
Fig. S10	page 18
Fig. S11	page 19
Fig. S12	page 20
Fig. S13	page 21
References	page 22

Experimental Section

Materials and Methods. All experiments were carried out under an N_2 atmosphere using standard Schlenk techniques and a glovebox. Acetonitrile was distilled over CaH_2 under an N_2 atmosphere. H_2 (99.9999%) was purchased from Sumitomo Seika Chemical Co., Ltd., Allyl chloride and bibenzyl were purchased from FUJIFILM Wako Pure Chemical Corporation, pyridine-2-carboxylic acid, 2,3-diaminopyridine, triphenyl phosphite, benzyl chloride, α-chloro-*p*-xylene, α-chloro-*o*-xylene, 1-(chloromethyl)naphthalene, cinnamyl chloride, 1,2-di(1-naphthyl)ethane, 1,5-hexadiene and *N*-*tert*-butyl-α-phenylnitrone (PBN) were purchased from Tokyo Chemical Industry Co. Ltd. and $CDCl_3$ and DMSO-d_6 was purchased from Cambridge Isotope Laboratories, Inc. These reagents were used without further purification.

Electrospray ionisation-mass spectrometry (ESI-MS) data were obtained by a JEOL JMS-T100LC AccuTOF. Proton nuclear magnetic resonance (^{1}H NMR) spectra were recorded on a Bruker Avance III 600 FT-NMR spectrometer. Chemical shifts in $CDCl_3$ and DMSO-d_6 were referenced to tetramethylsilane (TMS) for ^{1}H NMR. Ultraviolet-visible-near-infrared (UV-vis-NIR) spectra were recorded on a JASCO V-670 UV-visible-NIR spectrophotometer. Gas chromatography-mass spectrometry (GC-MS) data were recorded on an Agilent 7890B GC coupled to a 5977B MSD. X-ray photoelectron spectroscopy (XPS) spectra were recorded on an ULVAC PHI 5000 VersaProbe II system with an Al anode X-ray source. Binding energies were calibrated by the C 1*s* peak of carbon at 284.5 eV.[1] Photo-irradiation experiments were carried out using a light-emitting diode (LED), 400–800 nm, TLBC1-15X37-W (Aitec System Co., Ltd., Japan). Distance from the light source to the reaction media: 5 cm. Intensity of the light: 300 mW cm^{-2} (measured by power meter). X-ray diffraction (XRD) patterns were measured using an X-ray diffractometer (Rigaku SmartLab) with Cu Kα radiation generated at 45 kV and 200 mA {scan speed: 0.01° min^{-1} (2θ = 10–80°)}.

***N,N'*-Bis(2-pyridinecarboxamide)-2,3-pyridine (XH_2).** Triphenyl phosphite (6.8 g, 0.022 mol) was added to a pyridine solution (12 mL) of 2,3-diaminopyridine (1.2 g, 0.011 mol) and pyridine-2-carboxylic acid (2.8 g, 0.022 mol). The solution was stirred at 80 °C for 4 h and standing for overnight, whose volume was reduced to 6 mL by evaporation. Then, the solid was collected by filtration and washed with pyridine. The white powder was dried in vacuo {yield: 21% based on 2,3-diaminopyridine}. ^{1}H NMR (600 MHz, in $CDCl_3$,

referenced to TMS): δ 11.2 (s, 1H, –N*H*), 10.4 (s, 1H, –N*H*), 8.66 (d, 1H, –CO–NC$_5$*H*$_4$), 8.63 (d, 1H, –CO–NC$_5$*H*$_4$), 8.60 (dd, 1H, –N–NC$_5$*H*$_3$–N–), 8.40 (d, 1H, –CO–NC$_5$*H*$_4$), 8.30 (d, 1H, –N–NC$_5$*H*$_3$–N–), 8.25 (d, 1H, –CO–NC$_5$*H*$_4$), 7.94 (t, 1H, –CO–NC$_5$*H*$_4$), 7.86 (t, 1H, –CO–NC$_5$*H*$_4$), 7.52 (dd, 1H, –CO–NC$_5$*H*$_4$), 7.45 (dd, 1H, –CO–NC$_5$*H*$_4$), 7.34 (dd, 1H, –N–NC$_5$*H*$_3$–N–). Anal. Calcd for **XH$_2$**: $C_{17}H_{13}N_5O_2$: C, 63.94; H, 4.10; N, 21.93%. Found: C, 63.79; H, 4.13; N, 21.84%.

[H$^+$][Rh^{III}(X)(Cl)$_2$] {[H$^+$][1]}. A *N*,*N*-dimethylformamide (DMF) solution (12 mL) of *N*,*N'*-bis(2-pyridinecarboxamide)-2,3-pyridine (300 mg, 0.94 mmol) was added to a DMF solution (30 mL) of $Rh^{III}Cl_3$ (197 mg, 0.94 mmol). The solution was stirred at 100 °C for 1 h, then the solvent was removed under reduced pressure. The residue was washed with diethyl ether, acetonitrile and water, and the orange powder was collected by filtration {yield: 28% based on $Rh^{III}Cl_3$}. ^{1}H NMR (600 MHz, in DMSO-d_6, referenced to TMS): δ 14.6 (s, 1H, –N*H*–)), 9.42 (d, 1H, –CO–NC$_5$*H*$_4$), 9.36 (d, 1H, –CO–NC$_5$*H*$_4$), 9.17 (dd, 1H, –N–NC$_5$*H*$_3$–N–), 8.31 (t, 1H, –CO–NC$_5$*H*$_4$), 8.25 (t, 1H, –CO–NC$_5$*H*$_4$), 8.18 (dd, 1H, –CO–NC$_5$*H*$_4$), 8.04 (dd, 1H, –CO–NC$_5$*H*$_4$), 7.97 (m, 1H, –CO–NC$_5$*H*$_4$), 7.90 (d, 1H, –N–NC$_5$*H*$_3$–N–), 7.88 (m, 1H, –CO–NC$_5$*H*$_4$), 7.40 (dd, 1H, –N–NC$_5$*H*$_3$–N–). ESI-MS (in methanol): *m*/*z* 490.0 {[**1**]$^-$, relative intensity (*I*) = 100% in the range of *m*/*z* 200 to 2000}. Anal. Calcd for [H$^+$][**1**]•H$_2$O: $C_{17}H_{14}Cl_2N_5O_3Rh$: C, 40.03; H, 2.77; N, 13.73%. Found: C, 39.95; H, 2.64; N, 13.59%.

[H$^+$]$_2$[Rh^I(X)][PF$_6$] {[H$^+$]$_2$[2][PF$_6$]}. Complex **1** (10 mg, 0.020 mmol) was dissolved in H_2O (pH 10.0, 7.0 mL), and the solution was stirred at 60 °C for 8 h under a H_2 atmosphere (0.7 MPa) to form [H$^+$][**2**]. An aqueous solution of KPF_6 (10.5 mM, 4 mL) was added to the aqueous solution of [H$^+$][**2**]. Black precipitates were formed and collected by filtration. The black powder was washed by water and dried in vacuo {yield: 67% based on complex **1**}. ESI-MS (in methanol): *m*/*z* 421.9 {[**2**+2H]$^+$, *I* = 100% in the range of *m*/*z* 200 to 2000}. Anal. Calcd for [H$^+$]$_2$[**2**][PF$_6$]•0.5H$_2$O: $C_{17}H_{14}F_6N_5O_{2.5}RhP$: C, 35.44; H, 2.45; N, 12.15%. Found: C, 35.26; H, 2.48; N, 12.03%.

[Na$^+$][Rh^{III}(X)(CH$_2$C$_6$H$_5$)(Cl)] {[Na$^+$][3]}. All procedure was conducted under dark conditions. A benzyl chloride (23.6 μL, 0.21 mmol) was added to the aqueous solution of complex **2** (5.1 mM, 4.0 mL) that was prepared by the reaction of **1** with H_2 in an aqueous NaOH solution (pH 10.0). The resulting solution was stirred at room temperature overnight under an N_2 atmosphere. Then the solvent was removed under reduced pressure and the

orange powder was collected by filtration {yield: 88% based on **2**}. ^{1}H NMR (600 MHz, in $CDCl_3$, referenced to TMS): δ 8.89 (d, 1H, –N–NC_5H_3–N–), 8.59 (t, 2H, –CO–NC_5H_4), 8.14-8.10 (m, 2H, –CO–NC_5H_4), 8.08 (d, 1H, –N–NC_5H_3–N–), 7.97 (d, 2H, –CO–NC_5H_4), 7.62-7.58 (m, 2H, –CO–NC_5H_4), 7.02 (dd, 1H, –N–NC_5H_3–N–), 6.87(t, 1H, –CH_2–C_6H_5), 6.68 (t, 2H, –CH_2–C_6H_5), 6.39 (d, 2H, –CH_2–C_6H_5), 3.25 (dd, $J_{Rh–H}$ = 3.6 Hz, J_{gem} = 8.4 Hz, 1H, Rh–CH_2–), 3.21 (dd, $J_{Rh–H}$ = 3.6 Hz, J_{gem} = 8.4 Hz, 1H, Rh–CH_2–). ESI-MS (in methanol): *m*/*z* 512.0 {[**3**–Cl+H]$^+$, *I* = 100% in the range of *m*/*z* 200 to 2000}. Anal. Calcd for [Na^+][**3**]: $C_{24}H_{18}ClN_5NaO_2Rh$: C, 50.59; H, 3.18; N, 12.29%. Found: C, 50.89; H, 3.33; N, 12.27%.

1,2-Di(p-tolyl)ethane. Magnesium turnings (57.8 mg, 2.38 mmol) were added to a THF solution (5 mL) of α-chloro-*p*-xylene (625 µL, 4.75 mmol), which was stirred at 60 °C for 10 h. The solvent was removed under reduced pressure, and the residue was dissolved in methanol (20 mL). H_2O (20 mL) was added to the methanol solution to precipitate the product. White solid was collected by filtration and dried in vacuo {yield: 53% based on α-chloro-*p*-xylene}. ^{1}H NMR (400 MHz, in $CDCl_3$, referenced to TMS): δ 7.08 (s, 8H, CH_3–C_6H_4–CH_2–), 2.85 (s, 4H, –C_6H_4–CH_2–CH_2–), 2.32 (s, 6H, CH_3–C_6H_4–).

1-Methyl-2-[2-(2-methylphenyl)ethyl]benzene. Magnesium turnings (57.8 mg, 2.38 mmol) were added to the THF solution (5 mL) of α-chloro-*o*-xylene (613 µL, 4.75 mmol), which was stirred at 60 °C for 10 h. The solvent was removed under reduced pressure, and the residue was dissolved in methanol (20 mL). H_2O (20 mL) was added to the methanol solution to precipitate the product. White solid was collected by filtration and dried in vacuo {yield: 51% based on α-chloro-*o*-xylene}. ^{1}H NMR (400 MHz, in $CDCl_3$, referenced to TMS): δ 7.16–7.12 (m, 8H, CH_3–C_6H_4–CH_2–), 2.85 (s, 4H, –C_6H_4–CH_2–CH_2–), 2.32 (s, 6H, CH_3–C_6H_4–).

(1*E*,5*E*)-6-Phenylhexa-1,5-dienylbenzene (a) and (*E*)-Hexa-1,5-diene-1,4-diyldibenzene (b). Magnesium turnings (51.9 mg, 2.13 mmol) were added to the THF solution (5 mL) of cinnamyl chloride (592 µL, 4.27 mmol), which was stirred at 60 °C for 10 h. Hydrochloric acid (2 M, 5 mL) was added to the resulting solution, and the mixture was extracted with ethyl acetate (3 x 10 mL). The extract was washed with brine and dried with anhydrous sodium sulfate. Then, the solvent was removed under reduced pressure to give crude product. The residue was purified by silica gel flash column chromatography eluted by *n*-pentane/ethyl acetate (10/1) to give the

mixture of **a** and **b**. {yield: 44% based on cinnamyl chloride}. ^{1}H NMR (400 MHz, in $CDCl_3$, referenced to TMS): δ 7.35-7.16 (m, 10H, C_6H_5–CH_2–, **a** and **b** and C_6H_5–CH–, **b**), 6.44 (d, 2H, C_6H_5–C*H*=CH–, **a**), 6.38 (d, 1H, C_6H_5–C*H*=CH–, **b**), 6.28-6.26 (m, 2H, –CH=C*H*–CH_2–, **a**), 6.15-6.10 (m, 1H, –CH=C*H*–CH_2–, **b**), 6.05-6.01 (m, 1H, CH_2=C*H*–CHC_6H_5–, **b**), 5.08-5.04 (m, 2H, CH_2=CH–, **b**), 3.42 (q, 1H, =CH–C*H*C_6H_5–CH_2–, **b**), 2.66-2.62 (m, 2H, =CH–CH_2–CHC_6H_5–, **b**), 2.39 (t, 4H, =CH–CH_2–CH_2–, **a**).

Formation of Bibenzyl from the Reaction of 3 with Benzyl Chloride. Methanol was used for the solvent for this coupling reaction because both complex **3** and benzyl chloride dissolve to methanol. A methanol solution (1 mL) of **3** (2.8 mg, 5.1 mM) and benzyl chloride (29.4 µL, 256 µmol) was stirred under visible light at room temperature for 3 h. The resulting solution (400 µL) was added to diethyl ether (1 mL), which was passed through the silica gel column to remove complex(es). The products were analysed and quantified by GC-MS with mesitylene as an internal standard. The yield of bibenzyl was determined as 34% based on complex **3**.

The Reaction of 3 with Benzyl Chloride in the Presence of PBN. A methanol solution (1 mL) of **3** (2.8 mg, 5.1 mM), benzyl chloride (29.4 µL, 256 µmol) and PBN (453 mg, 2.56 M) was stirred under visible light at room temperature for 3 h. The resulting solution (400 µL) was added to diethyl ether (1 mL), then which was passed through the silica gel column to remove complex(es). The products were analysed and quantified by GC-MS with mesitylene as an internal standard.

General Procedure for Coupling Reaction of Benzyl Chloride derivatives or Allyl Chlorides Derivatives. Ethanol was used for the solvent for the catalytic reactions to carry out the reactions at 80 °C. Benzyl chloride derivatives or allyl chloride (0.51 mmol), H_2O (51 µmol) and sodium acetate (417 mg, 5.1 mmol) were added to the ethanol solution (50 mL) of **1** (10 mol%), which was stirred with visible light at 80 °C for 12 h under a H_2 atmosphere (0.9 MPa). The catalytic reactions were stopped by the removing of the light source, and the reaction solution was kept in the dark during purification process. After the resulting solution was cooled to room temperature, the solvent was removed under reduced pressure. The residue was extracted with diethyl ether (5 x 5 mL), and the solvent was removed under reduced pressure. The solution was passed through a silica gel column eluted with *n*-hexane. After removing the solvent from the solution, the product was identified by ^{1}H NMR in $CDCl_3$ and GC-MS. For the isolation of

1,2-di(1-methylnaphthyl)ethane (entry 4), the white solid that was obtained after the silica gel column was washed with *n*-hexane and dried in vacuo. The isolated yields (isolated amounts) of products measured by a balance were 33% (15 mg) in entry 1, 30% (16 mg) in entry 2, 28% (15 mg) in entry 3, 34% (25 mg) in entry 4 and 5.6% (3.3 mg) for branched isomer and 3.9% (2.3 mg) for linear isomer in entry 6 (Table 1). No coupling products were formed without **1**, H_2 or photo-irradiation.

Green Chemistry Metric Calculations (E-factor) to Quantify the Waste and Compare with the Reported Methods. E-factors were calculated by the following equation: {(weight of reactants, reagents and solvents) – (weight of product)} / weight of product.[2] E-factors of previously reported reactions are 18.6 and 5.23.[3,4] E-factor of this work is 2602.

Isolation of (1*E*,5*E*)-6-Phenylhexa-1,5-dienylbenzene (a) and (*E*)-Hexa-1,5-diene-1,4-diyldibenzene (b) from the Coupling Reaction of Cinnamyl Chloride. Cinnamyl chloride (0.51 mmol), H_2O (51 μmol) and sodium acetate (417 mg, 5.1 mmol) were added to the ethanol solution (50 mL) of **1** (10 mol %), which was stirred with visible light at 80 °C for 12 h under a H_2 atmosphere (0.9 MPa). After the resulting solution was cooled to room temperature, the solvent was removed under reduced pressure. The residue was extracted with diethyl ether (5 x 5 mL), and the solvent was removed under reduced pressure. The solution was passed through a silica gel column eluted with *n*-hexane. After removing the solvent from the solution, the mixture of **a** and **b** was added to *n*-hexane (300 μL). The *n*-hexane solution was passed through a $AgNO_3$ (20%)-silica gel column eluted with *n*-hexane/*n*-butyl ethyl ether (97/3).[5] The solvent was removed under reduced pressure to yield white powders for **a** and colourless oil for **b** {yield: 3.9% for **a** and 5.6% for **b**}.

Quantitative Analysis of 1,5-Hexadiene by GC-MS. Allyl chloride (0.51 mmol) and sodium acetate (417 mg, 5.1 mmol) were added to the ethanol solution (50 mL) of **1** (10 mol %), which was stirred with visible light at 80 °C for 12 h under H_2 atmosphere (0.9 MPa). The resulting solution (400 μL) was added to diethyl ether (1 mL), then which was passed through the silica gel column to remove complex(es). The products were analysed and quantified by GC-MS with mesitylene as an internal standard.

pH adjustment. pH values of aqueous solutions were adjusted by using an aqueous NaOH solution for pH 10 to 11. In a pH range of 10 to 11, the pH values of the solutions

were determined by a pH meter (IQ Scientific Instruments Inc., IQ200) equipped with a stainless steel micro pH probe (IQ Scientific Instruments Inc., PH15-SS).

X-ray Crystallographic Analyses of 1 and 3. An orange crystal of [PPh_4^+][**1**] was obtained from an acetonitrile/DMF (5/1) solution diffused with diethyl ether at room temperature in the presence of PPh_4Cl. An orange crystal of [H^+][**3**] was obtained from its acetonitrile solution diffused with diethyl ether under an N_2 atmosphere. Measurements were made on a Rigaku XtaLAB P200 with confocal monochromated Mo Kα radiation (λ = 0.7107 Å). Data were collected and processed using the CrystalClear program. All calculations were performed using the CrystalStructure crystallographic software package. Crystallographic data for **1** and **3** have been deposited with the Cambridge Crystallographic Data Centre as Supplementary Publication Nos. 2118319 (complex **1**) and 2118320 (complex **3**). Copies of the data can be obtained free of charge on application to CCDC, 12 Union Road, Cambridge CB2 1EZ, UK {fax: (+44)1223-336-033; e-mail: deposit@ccdc.cam.ac.uk}.

Table S1. Reductive C-C Coupling

Entry	Organic halide, Organic pseudohalide, or Olefine	Reductant	Solvent	Temp. (°C)	Yield (%)	Ref
1	Alkyl-X<br>sp^3-sp^3	Na	neat	0	< 58	[6]
2	Aryl-X, Alkyl-X<br>sp^2-sp^3	Na	ether	r.t.	< 60	[7]
3	Aryl-X<br>sp^2-sp^2	Cu	neat	200-285	< 81	[8]
4	Aryl-X<br>sp^2-sp^2	Zn	*N*,*N*-dimethylformamide	50	< 89	[9]
5	Aryl-X<br>sp^2-sp^2	H_2	H_2O/*n*-BuOH	30-50	< 50	[10]
6	alkene, benzoic anhydride<br>sp^2-sp^2	H_2	2-methoxyethyl ether	65	< 71	[11]
7	Aryl-X<br>sp^2-sp^2	H_2	H_2O	90-120	< 79	[12]
8	Aryl-X<br>sp^2-sp^2	*n*-Bu_4NBr<br>(*i*Pr)$_2$EtN	*p*-xylene	130	< 100	[13]
9	aldehyde, enone<br>sp^2-sp^2	H_2	dichloroethane	25	< 92	[14]
10	Aryl-X<br>sp^2-sp^2	H_2	neat	r.t.	< 17	[15]
11	Aryl-X, Alkyl-X<br>sp^2-sp^3	Mn	1,3-dimethyl-3,4,5,6-tetra-hydro-2(1*H*)-pyrimidinone	60-80	< 88	[16]
12	Alkyl-X<br>sp^3-sp^3	Zn	*N*,*N*-dimethylacetamide	25	< 92	[17]
13	Aryl-X, Aryl-OTf<br>sp^2-sp^2	Zn	*N*,*N*-dimethylformamide	40	< 94	[18]
14	Ar-X, alkyne<br>sp^3-sp	H_2	toluene	100	< 76	[3]
15	Allyl-NR_2, Allyl-OH, Allyl-OR,<br>sp^3-sp^3	H_2	*N*-methyl-2-pyrrolidone	80	< 96	[4]
16	Benzyl-X<br>Allyl-X<br>sp^3-sp^3	H_2	ethanol/H_2O	80	< 45	this work

Table S2. Characterisation of Rh complexes **1**, **2** and **3**

	**1**	**2**	**3**
X-ray	Fig. 1	-	Fig. 3
ESI-MS	Fig. S2	Fig. 2	Fig. S6
^{1}H NMR	Fig. S1	-	Fig. S7
UV-vis-NIR	Fig. S4	Fig. S4	Fig. S8
XPS	Fig. S5	Fig. S5	-

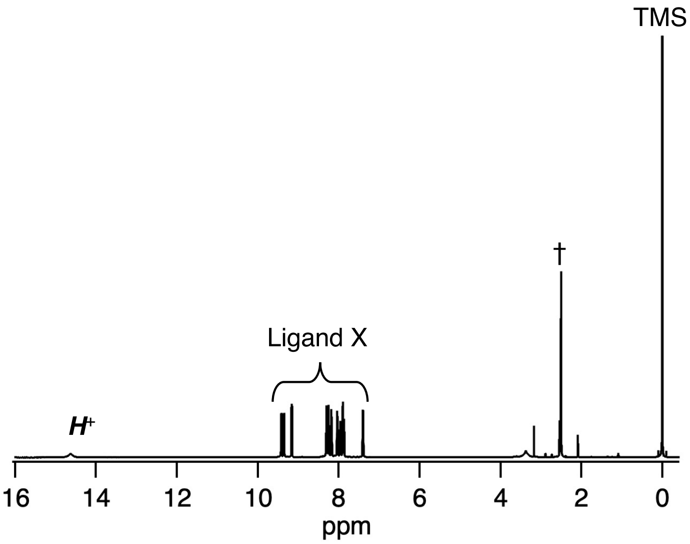

Figure S1. NMR spectrum of **1** in $CDCl_3$. Tetramethylsilane (TMS), reference with the methyl proton resonance set at 0.00 ppm. †: Peak of dimethylsulfoxide.

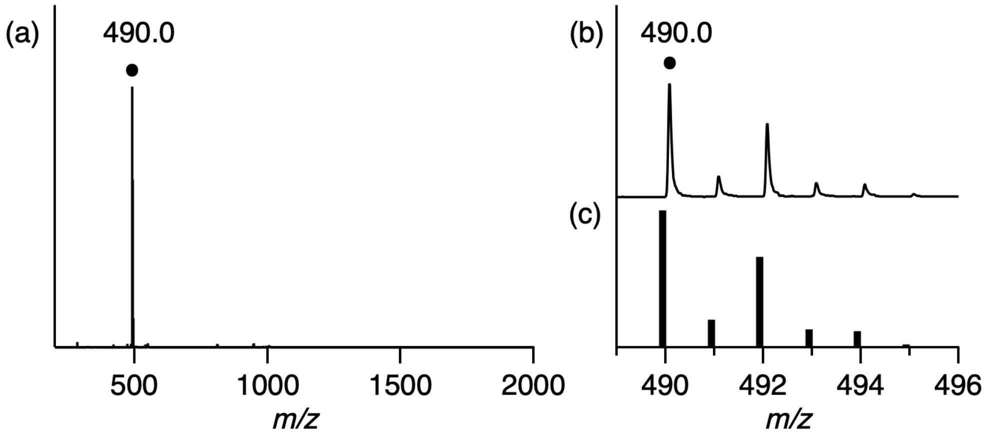

Figure S2. (a) Negative-ion ESI mass spectrum of **1** in methanol. The signal at m/z = 490.0 corresponds to [**1**]$^{-}$. (b) The signal at m/z = 490.0 for [**1**]$^{-}$. (c) The calculated isotopic distribution for [**1**]$^{-}$.

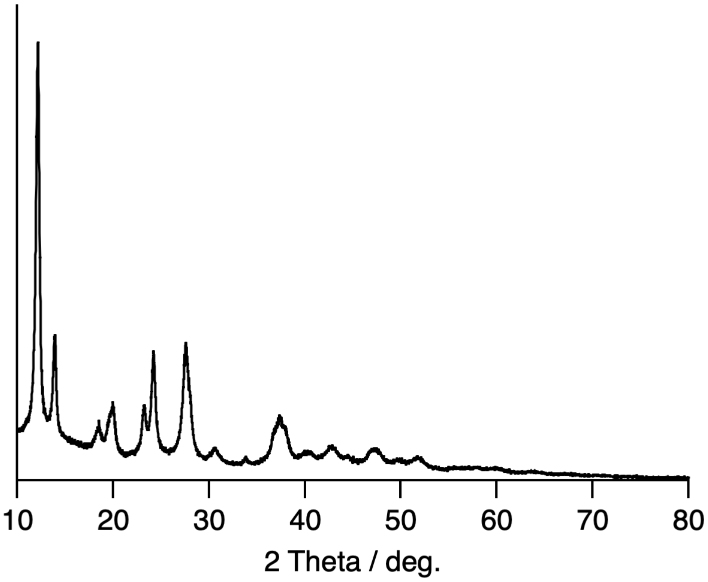

Figure S3. XRD pattern of complex **1**.

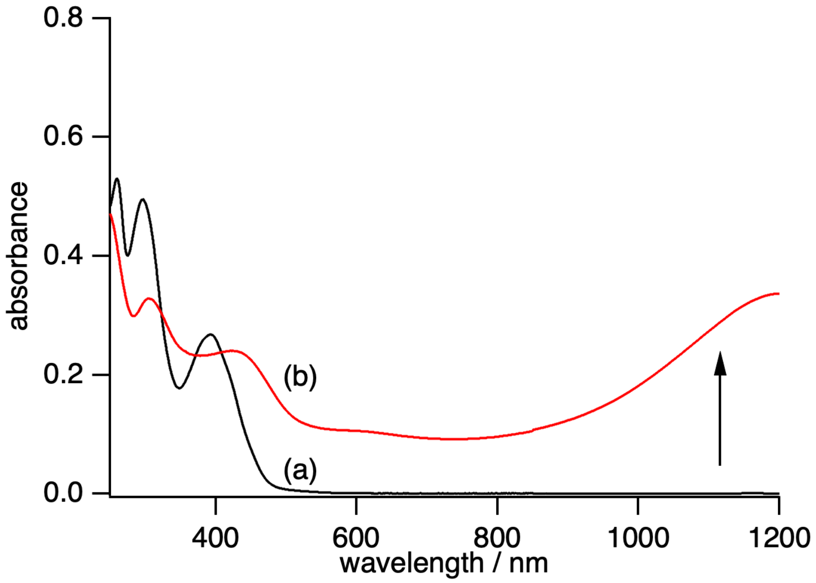

Figure S4. UV-vis-NIR absorption spectra of **1** (0.32 mM) (a) before H_2 and (b) after H_2 in H_2O (pH 11). Light pass length is 0.1 cm.

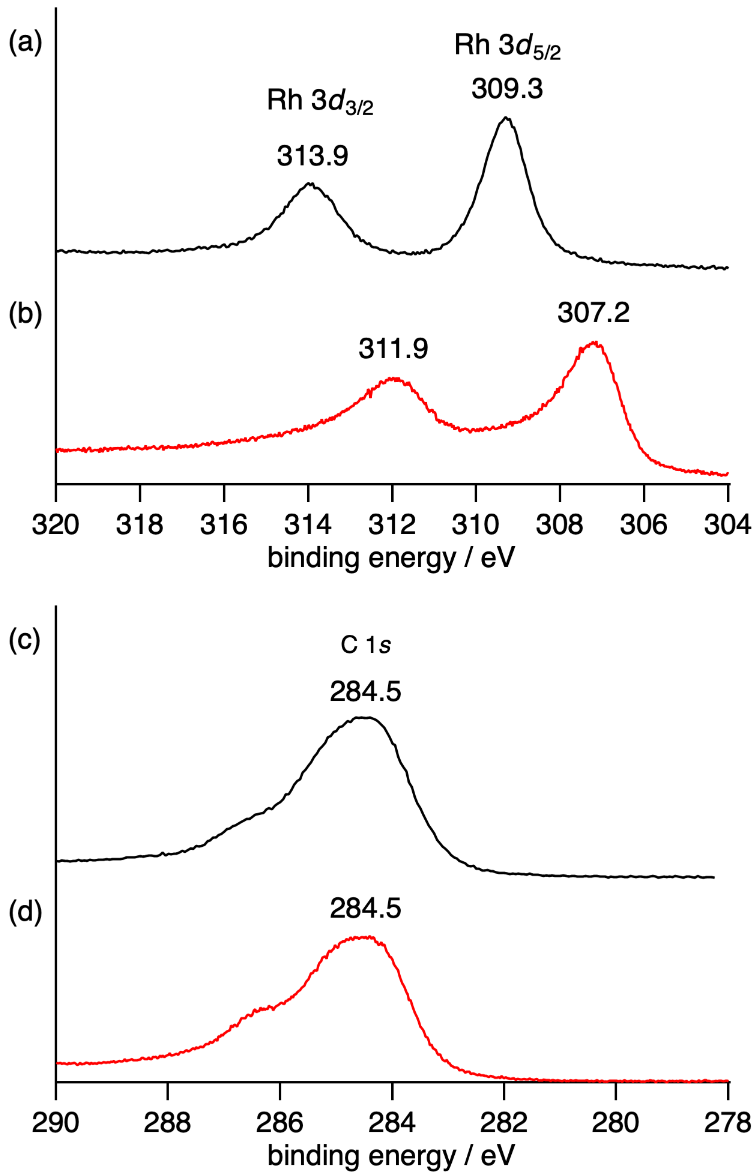

Figure S5. XPS spectra of the Rh3*d* region for (a) Rh^{III} complex **1** and (b) Rh^{I} complex **2** and the C1*s* region for (c) Rh^{III} complex **1** and (d) Rh^{I} complex **2**.

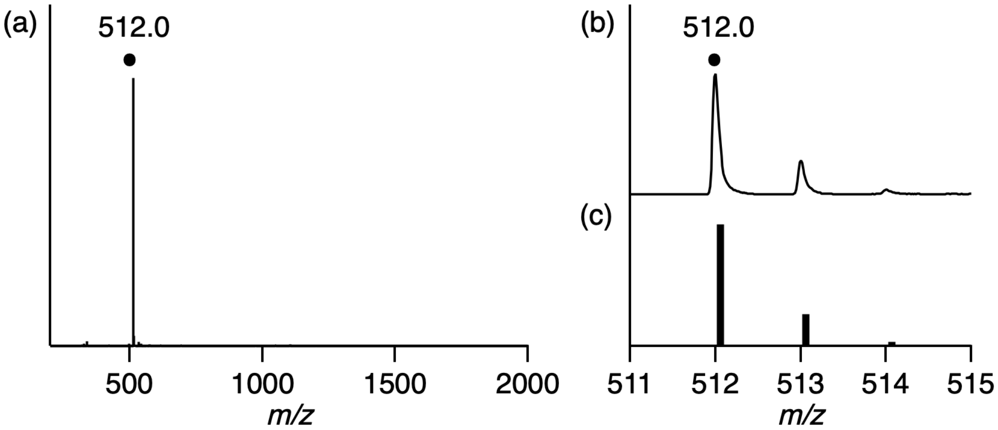

Figure S6. a) Positive-ion ESI mass spectrum of **3** in methanol. The signal at *m*/*z* 512.0 corresponds to [**3**–Cl+H]$^+$. b) The signal at *m*/*z* = 512.0 for [**3**–Cl+H]$^+$. c) The calculated isotopic distribution for [**3**–Cl+H]$^+$.

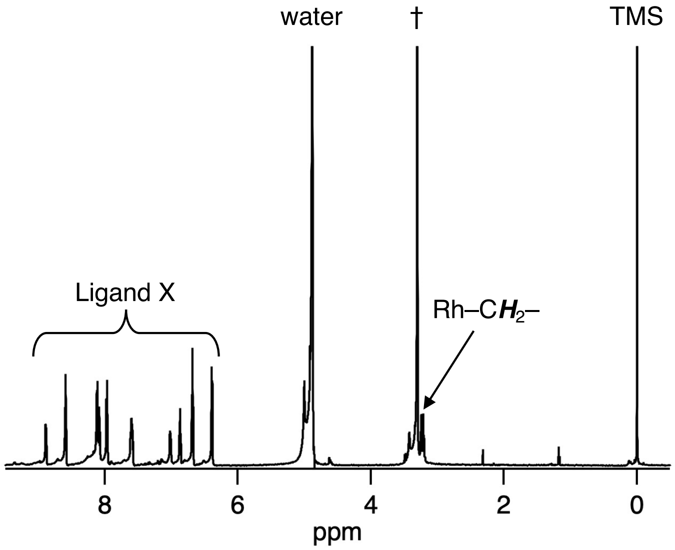

Figure S7. NMR spectrum of **3** in CD_3OD. Tetramethylsilane (TMS), reference with the methyl proton resonance set at 0.00 ppm. †: Peak of methanol.

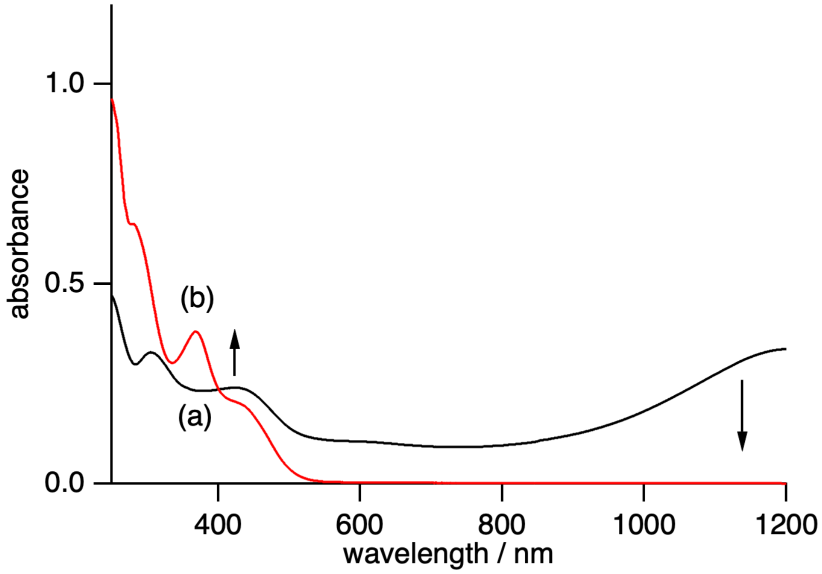

Figure S8. UV-vis-NIR absorption spectra of **2** (0.32 mM) in H_2O (a) before and (b) after addition of benzyl chloride. Light pass length is 0.1 cm.

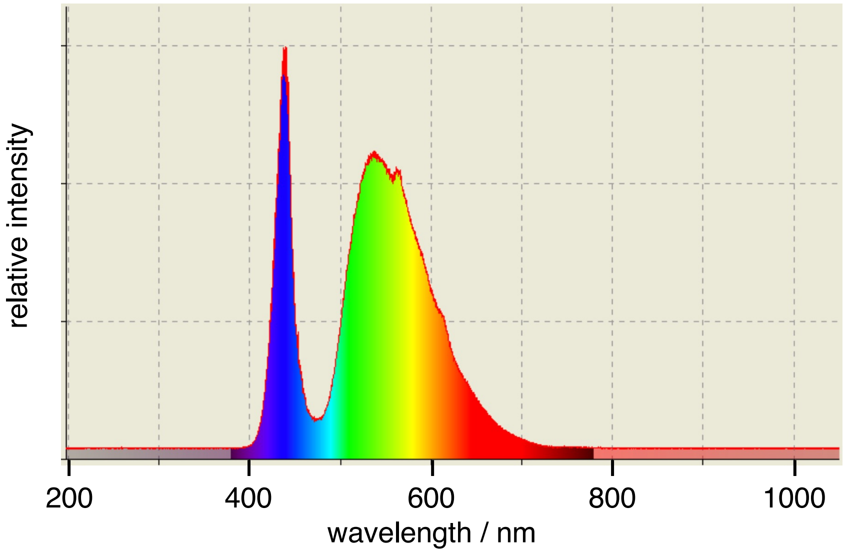

Figure S9. Emission spectrum of LED (TLBC1-15X37-W, Aitec System Co., Ltd.) using for the visible (400–800 nm) light irradiation experiments.

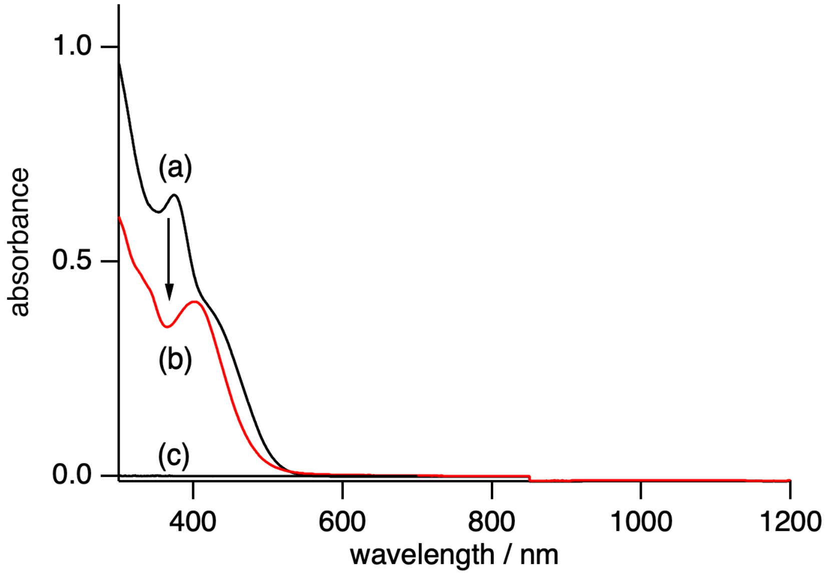

Figure S10. UV-vis-NIR absorption spectra of (a) the methanol solution of **3** (0.8 mM) and benzyl chloride, (b) the methanol solution of **3** (0.8 mM) and benzyl chloride after photo-irradiation and (c) methanol without **3** and benzyl chloride. It was confirmed that the methanol used had no absorption in this region. Light pass length is 0.1 cm.

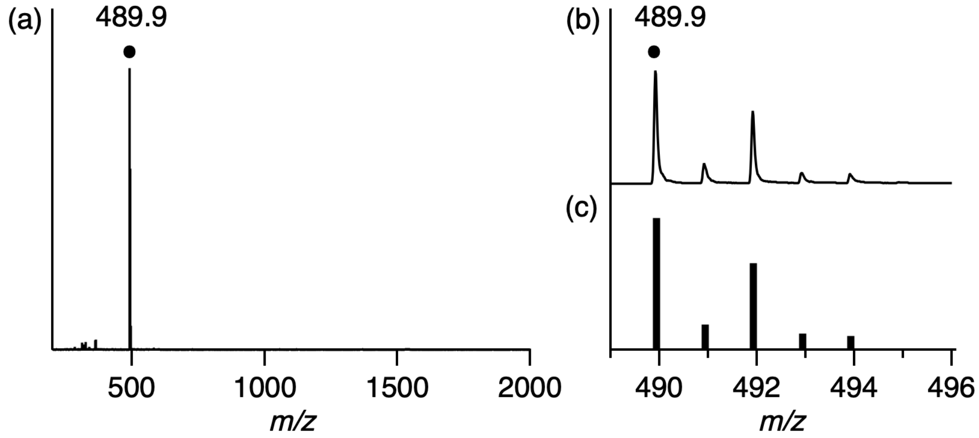

Figure S11. a) Negative-ion ESI mass spectrum of the reaction solution of **3** and benzyl chloride after photo-irradiation. The signal at *m*/*z* 489.9 corresponds to [**1**]$^{-}$. b) The signal at *m*/*z* = 489.9 for [**1**]$^{-}$. c) The calculated isotopic distribution for [**1**]$^{-}$.

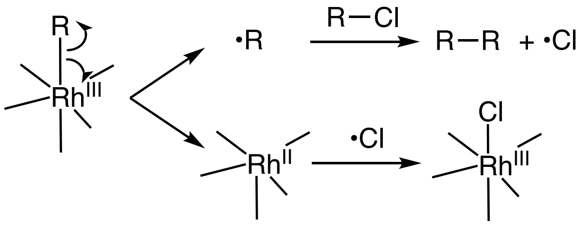

Figure S12. Proposed mechanism of the formation of bibenzyl from the reaction of **3** with R–Cl under photo-irradiation.

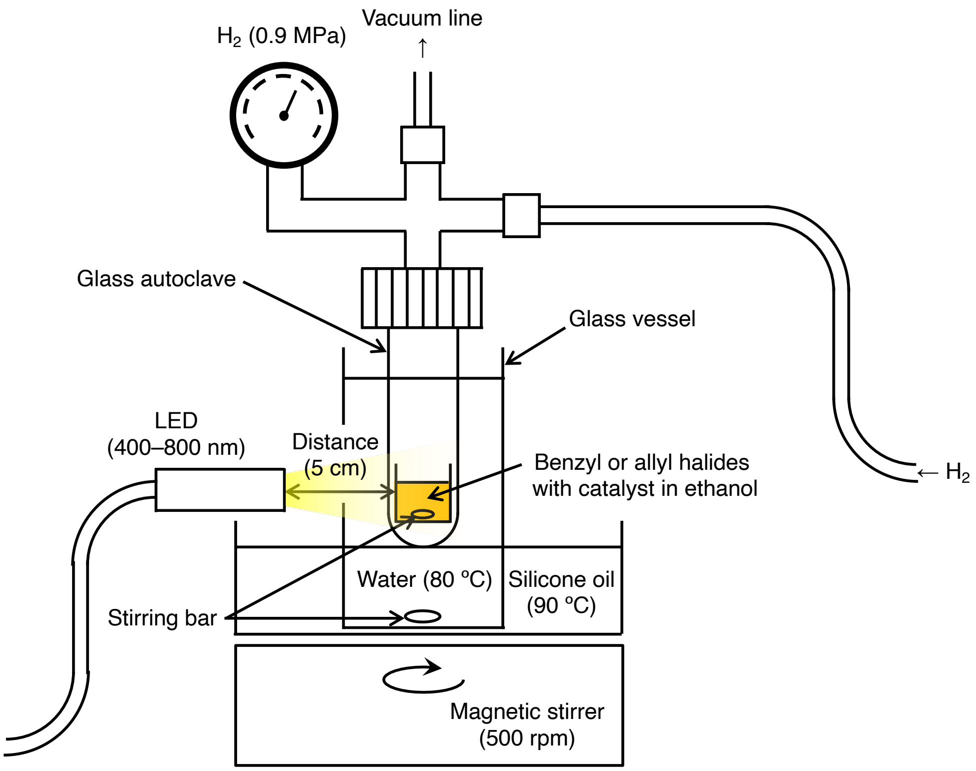

Figure S13. Reaction apparatus for the catalytic reductive coupling reaction under photo-irradiation. Time for photo-irradiation: 12 h. Distance from the light source to the reaction media: 5 cm. Temperature: 80 °C. Intensity of the light: 300 mW cm^{-2}.

References

[1] J. F. Moulder, W. F. Stickle, P. E. Sobol and K. D. Bomben, in *Handbook of X-ray Photoelectron Spectroscopy*; Chastin, J., King, R. C. Jr., Eds.; Physical Electronics, Inc.: Eden Prairie, MN, 1995, pp 40.

[2] R. A. Sheldon, *Chem. Commun.*, 2008, 3352.

[3] K. Semba, R. Kameyama and Y. Nakao, *Chem. Lett.,* 2018, **47**, 213.

[4] X. Zhou, G. Zhang, R. Huang and H. Huang, *Org. Lett.,* 2021, **23**, 365.

[5] A. F. Barrero, M. M. Herrador, J. F. Quilez del Moral, P. Arteaga, J. F. Arteaga, H. R. Diéguez and E. M. Sánchez, *J. Org. Chem.,* 2007, **72**, 2988.

[6] (a) A. Wurtz, *Ann. Chim. Phys.,* 1855, **44**, 275; (b) A. A. Morton, J. B. Davidson and B. L. Hakan, *J. Am. Chem. Soc.,* 1942, **64**, 2242.

[7] (a) B. Tollens and R. Fittig, *Ann. Chem. Pharm.,* 1864, **131**, 303; (b) Z. Wang, *Comprehensive Organic Name Reactions and Reagents*, Wiley, Hoboken, 2010, pp. 3100.

[8] F. Ullmann and J. Bielecki, *Chem. Ber.*, 1901, **34**, 2174.

[9] M. Zembayashi, K. Tamao, J. Yoshida and M. Kumada, *Tetrahedron Lett.,* 1977, **18**, 4089.

[10] D. V. Davydov and I. P. Beletskaya, *Russ. Chem. Bull.,* 1995, **44**, 1139.

[11] K. Kokubo, M. Miura and M. Nomura, *Organometallics,* 1995, **14**, 4521.

[12] S. Mukhopadhyay, G. Rothenberg, H. Wiener and Y. Sasson, *Tetrahedron*, 1999, **55**, 14763.

[13] J. Hassan, C. Hathroubi, C. Gozzi and M. Lemaire, *Tetrahedron*, 2001, **57**, 7845.

[14] H. Y. Jang, R. R. Huddleston and M. J. Krische, *J. Am. Chem. Soc.,* 2002, **124**, 15156.

[15] K. Ma, W. E. Piers and M. Parvez, *J. Am. Chem. Soc.,* 2006, **128**, 3303.

[16] D. A. Everson, R. Shrestha and D. J. Weix, *J. Am. Chem. Soc.,* 2010, **132**, 920.

[17] X. Yu, T. Yang, S. Wang, H. Xu and H. Gong, *Org. Lett.*, 2011, **13**, 2138.

[18] L. K. G. Ackerman, M. M. Lovell and D. J. Weix, *Nature*, 2015, **524**, 454.
